# Risky Sexual Practice and Associated Factors Among Youth Preparatory Students in Gondar City, Northwest Ethiopia

**DOI:** 10.3389/fpubh.2022.843359

**Published:** 2022-05-02

**Authors:** Zelalem Nigussie Azene, Lanchisl Tsegaye, Mekdes W/Gebriel, Adamu Tadesse, Abreham Tadele, Getie Lake Aynalem, Zewudu Andualem, Birhan Tsegaw Taye

**Affiliations:** ^1^Department of Women's and Family Health, School of Midwifery, College of Medicine and Health Sciences, University of Gondar, Gondar, Ethiopia; ^2^Department of Midwifery, College of Medicine and Health Sciences, Dilla, Ethiopia; ^3^Department of Clinical Midwifery, School of Midwifery, College of Medicine and Health Sciences, University of Gondar, Gondar, Ethiopia; ^4^Department of Environmental and Occupational Health and Safety, Institute of Public Health, College of Medicine and Health Sciences, University of Gondar, Gondar, Ethiopia; ^5^School of Nursing and Midwifery, Asrat Woldeyes Health Science Campus, Debre Berhan University, Debre Berhan, Ethiopia

**Keywords:** risky sexual practice, preparatory school, students, youth, Ethiopia

## Abstract

**Background:**

Risky sexual practices can negatively affect the health of youths by predisposing them to a variety of sexually transmitted infections, including HIV/AIDS and unwanted pregnancy, which, in turn, would lead to serious lifelong deleterious health, social, and economic consequences. While youths tend to be less well-informed and require more information, little has been known in Ethiopia. Therefore, this study aimed to assess the prevalence of risky sexual practices and associated factors among youth students in Gondar city, northwest Ethiopia.

**Methods:**

A cross-sectional study was conducted among 414 regularly attending youth students in Gondar city from April to May 2019. A simple random sampling technique was used to select the study participants. Data were collected by using a pre-tested, structured, and self-administered questionnaire. Bivariable and multivariable logistic regression analyses were employed, and a multivariable binary logistic regression model was used to identify the effect of independent variables on the outcome variable at *p* < 0.05 with its 95%CI.

**Result:**

The prevalence of risky sexual practices was 49.3%. Peer pressure (AOR = 1.99, 95%CI: 1.21, 3.26), drinking alcohol (AOR = 4.88 95%CI: 3.06, 7.79), and watching pornography (AOR = 2.82, 95%CI: 1.74, 4.56) were positively associated with the risky sexual practice of youths. Whereas, age, gender, and pocket money did not have any association with risky sexual practice in this study.

**Conclusion:**

In this study, the prevalence of risky sexual practices was found to be high. Thus, multisector collaboration efforts are needed from parents, schools, health facilities, and the government to tackle the exposure of in-school youth toward peer pressure, drinking alcohol, and watching pornographic films, which in turn helps to bring about healthy sexual practices among them.

## Introduction

Risky sexual practices are any sexual activity that increases the risk of contracting sexually transmitted infections (STI) and unintended pregnancy, which includes having sex with multiple sexual partners, early initiation of sexual intercourse under the age of 18, and not using or inconsistent use of a condom (1, 2). Based on different sources, the term youth refers to the age interval between 15 and 24 years old, and since people become sexually active at this age, a healthy sexual awareness and development class is mandatory for the future health status of the youths and adolescents in particular (3, 4).

Young people aged 10–24 years constitute around 1.8 billion and represent 27% of the world's population (5). Youths comprise one-third of Ethiopia's population, belonging to the age category of 15–24 years (6). Adolescence and youth are critical developmental periods when they begin to define and clarify their sexual values and start to experiment with sexual behaviors. Most of these youths are students, and they are also at high risk for unsafe sexual practices and problems like HIV/AIDS or STI, unwanted pregnancy, abortion, poor school performance, high school dropout rate, psycho-social problems, conduct disorder, divorce, and economic problems (7, 8).

Youths are defined as those between the ages of 15 and 24 years, which is considered a period of transition from childhood to adulthood (i.e., from the dependence of childhood to adulthood's independence) (9). They are engaged in high-risk behaviors like smoking cigarettes, drinking alcohol, use of drugs, suicide, unprotected sexual practice, unintended pregnancy, and violence (10–12). These behaviors and lifestyles learned or adopted during this period will influence health both in the present and in the future. Thus, important life-long health habits are established and carried into adulthood, in turn leading them to take part in sexual risk practices (13).

Adolescents and youths are not only a time of tremendous opportunity and change but also a time of heightened vulnerability to a variety of sexual, reproductive, social, emotional health risks including unwanted pregnancy, STI/HIV/AIDS, abortion, detachment from families, discontinuation of schooling, depression, and streetism (14). The trend of STI and HIV prevalence over time was higher in Gondar and Bahir Dar cities compared with major cities of Ethiopia (15).

Youth people's vulnerability to risky and other unhealthy behaviors is tied to a host of individual, family, and community factors that influence their behavior and that are closely related to their economic and educational opportunities (16–18).

One-third of the 340 million new STIs cases occur per year in people under 25 years of age worldwide. According to the United Nations Program on HIV/AIDS (UNAIDS), in 2008, young people aged 15–24 years accounted for 42% of new HIV infections, and nearly 80% of these individuals live in sub-Saharan Africa (19). Results from the 2015 USA national youth risky behavior surveillance system (YRBSS) indicated that many high school students are engaged in priority health-risk behaviors associated with the leading causes of death among persons aged 10–24 years. During the 12 months before the study, many high school students are engaged in sexual risk practices resulting in unintended pregnancies and STIs, including HIV infection. Nationwide, 41.2% of students had ever had sexual intercourse, 30.1% had had sexual intercourse during the 3 months before the survey, and 11.5% had had sexual intercourse with four or more persons during their life (20). In Ethiopia, a systemic review and meta-analysis study showed that the pooled prevalence of risky sexual practice was 42.80% (21).

Youth health efforts should focus on prevention since most of the disease burden is preventable, and prevention is a cost-effective strategy concerning adolescents (22). Former studies determined that chat chewing, social media, absence of interpersonal support, relationship stress, poor mother-daughter attachment, intimate partner violence, lack of religious involvement, drug and alcohol use, and treating a physical problem with prescription drugs can influence the practice of risky sexual practice of youth (21, 23–25). Local organizations have been supporting activities to increase access to sexual and reproductive health (RH) services for young people in all schools. This includes the establishment of sexual and reproductive health rights clubs in the school and the scaling-up and institutionalization of youth-friendly services through capacity building at all levels of the health system. Nevertheless, the effects of all these efforts have not been well implicit across the Ethiopian high schools and preparatory schools (26).

Preparatory school students are assets of the society and change agents in filling the gap in the past and on whom the future generation is based. It is also clear that this segment of the population is on the way to transforming into an adulthood filled with ambition and building their future academic and social career, neglecting their sexual and reproductive health can cause high social and economic costs, both immediately and in the year ahead. Existing limitations in Ethiopia were being tailored to examine the role of a single variable on the sexual behavior of youth like parenting practices, peer influence, substance use, and living arrangement separately and also being concentrated among university and college students. However, preparatory students are nested in a context where many of the aforementioned factors interact. Therefore, this study aimed to assess the prevalence of risky sexual practices and associated factors among preparatory school students in Gondar city, northwest Ethiopia.

## Methods

### Study Design, Period, and Setting

A cross-sectional study was conducted from April to May 2019 in Gondar city. The city is located in Central Gondar Zone, Amhara Regional State of Ethiopia and is about 748 km northwest of Addis Ababa, the capital of Ethiopia, and about 180 kilometers from Bahir Dar city, the capital of Amhara regional state. The city has an estimated total population of 324,000. It has an altitude of 12°36′N 37°28′E and a longitude of 12.60N 37.467'E with an elevation of 2,133 m above sea level and is divided into 12 administrative areas (sub-cities), which consist of 21 kebeles (the smallest administrative units). Gondar is among one of the ancient and largely populated cities in the country. The city has 11 governmental preparatory schools and 4 private preparatory schools. All these schools, currently providing educational services, were included in the study. This study was conducted at seven randomly selected preparatory schools (i.e., five governmental and two private).

### Participants

All youths aged between 15 and 24 years who attend Gondar city preparatory schools (grades 11 and 12) were the source population and those who attended the selected schools were the study population, and students available during the data collection period were included in the study.

### Inclusion and Exclusion Criteria

Those students who were available and attending class during the data collection period were included, whereas those who were seriously ill and were unable to respond and night and extension students were excluded from this study.

### Sample Size Determination

The required sample size of eligible students for the study was calculated using the formula to estimate a single population proportion. The following assumptions were made to calculate the sample size: (a) A 95% probability of obtaining the population proportion of preparatory school students who experienced risky sexual practices within a 5% margin of error and (b) based on a study conducted in Ethiopia (7), the population proportion of preparatory school students who had risky sexual behaviors was assumed to be 42.8% (21).


(1) $$n\;=\;\frac{{\left(Z\alpha /2\right)}^{2}p(q)}{{\left(w\right)}^{2}}\;=\;\frac{{\left(1.96\right)}^{2}*0.428(0.572)}{(0.05)2}\;=\;376.19$$

Therefore, the required sample size was 376. Expecting a 10% non-response rate, the final sample size was calculated to be 414.

### Sampling Technique and Procedure

Seven preparatory schools (i.e., five governmental and two private) were randomly selected from all 15 preparatory schools. The lists of students were obtained from each selected school registrar's office, and the sampling frame was designed by numbering the list of students. Then, the total sample size was distributed to each school based on proportional allocation to their size by using the proportional allocation formula ($ni=\frac{Ni}{N}*n$). Finally, students from each selected school in each class were selected by a simple random sampling technique using a table of random generation.

### Dependent Variable

Risky sexual practices include multiple sexual partners, early initiation of sex, failure to use condoms, and sex with commercial sex workers.

### Explanatory Variables

Sociodemographic variables include age, sex, religion, attending night club, watching pornographic video, psychosocial factors, the type of social media used, and substance/drug use.

### Measurements

Risky sexual practices were considered as at least one of those that a student is involved in: multiple sexual partners (having more than one sexual partner until the survey), early initiation of sex (sexual debut at the age of <18 years of age), condom (inconsistent use of/failure to use the condom at least ones during sexual intercourse until the survey), sex with commercial sex workers (at least once until the survey) (27).

Early sexual initiation was defined as the experience of sexual intercourse before the age of 18 years (28).

Youth was defined as a part of the population who are in the age group of 15–24 years old (9).

Sexually active was considered for students who claimed to engage in a sexual act at least once before the study (28).

Multiple sexual partners involves having two and above sexual partners in their lifetime.

Commercial sex worker is defined as a person who works in the adult entertainment industry characterized by the provision of sexual favors for financial and non-financial rewards with a varying degree of physical contact between the parties.

Living arrangement is defined as the state of students' living with their parents, relatives, husband or wife, or others living with them.

Peer pressure, according to this study, is defined as respondents who are going to be under peer pressure if they experience any influence from their friends to have sex.

Substance use is defined as any use of at least any one of the following substances: alcohol, Chat, cigarette, Shisha, Hashish, or drugs that are assumed to affect levels of thinking and an increased risk of involving in risky sexual behavior (1).

### Data Collection Procedures

A pretested and structured self-administered questionnaire was used to collect the data from the study participants. This questionnaire comprises four items namely, sociodemographic factors, psychosocial factors, type of social media users, and substance/drug use. The questionnaire was first prepared in English and then translated to the local language (i.e., Amharic) and back to English to maintain consistency of the tool. Data were collected by four diploma-holder midwives. A self-administered questionnaire was used to collect data from all selected preparatory school students who consented to be a part of the study. A one-day training was provided for the four diploma-holder midwives and one BSc midwife for supervision about techniques of data collection. The principal investigator and supervisor made day-to-day on-site supervision during the whole period of data collection and checked each questionnaire daily for completeness and consistency. The questionnaire was pre-tested to check the response, language, clarity, and appropriateness of the questionnaire while the pretest was done outside the study area with 5% of sample size, i.e., on 20 students. Based on the findings of the pre-test, modification of the questionnaire was done and questions were revised accordingly. The overall data collection process was supervised by the principal investigator.

### Data Processing and Analysis

The data were first checked manually for completeness and then coded and entered into Epi Info version 7.1.2 (29). Then, the data were exported to Statistical Package of Social Science (SPSS) version 25 (30) for data checking, cleaning, and analysis. Descriptive statistics (like mean, standard deviation, frequencies, and percentages) were used to describe the study population about dependent and independent variables. Results were presented in text, tables, graphs, and charts.

Binary logistic regression (bivariable and multivariable logistic regression) was used to identify statistically significant independent variables, and variables having a *p-*value <0.2 in the bivariable analysis were entered into multivariable logistic regression for further analysis. A *p-*value <0.05 in the multivariable analysis was considered significant. Hosmer–Lemeshow goodness-of-fit was used to test the model fitness. Adjusted odds ratio (AOR) with a 95% confidence interval was used to identify factors associated with risky sexual practice among students.

## Result

### Sociodemographic Characteristics of the Study Participants

A total of 414 students participated with an overall response rate of %. Of these 234 (56.5%) were women. The mean age of the respondents was 19 years ± 1.61 SD. The majority, 339 (82.9%) of the study participants were orthodox by religion and 347 (83.8%) belong to Amhara ethnic group. Respondents who were living with parents account for 253 (61.1%). About, 245 (59.2%) were grade 11 students. The mean family monthly income was 4,967 ETB ± 2,206 SD (Table 1).

**Table 1:** Sociodemographic characteristics of study participants and their parents in Gondar city, Northwest Ethiopia, 2019 (*n* = 414).

**Variables**		**Frequency**	**Percentage**
Age in years	15–18	210	50.70
	19–24	204	49.30
Sex	Male	180	43.50
	Female	234	56.50
Grade level	Grade11	245	59.20
	Grade12	169	40.80
Religion	Orthodox	308	74.40
	Muslim	63	15.20
	Protestant	37	8.90
	Catholic	6	1.50
Religious attendance	Yes	306	73.90
	No	108	26.10
Frequency Religious attendance (*n =* 306)	Daily	95	22.90
	Most of the time	68	16.40
	Once a week	122	29.50
	Once a month	21	5.20
Ethnicity	Amhara	347	83.80
	Tigray	26	6.30
	Qimant	26	6.30
	Oromo	13	3.10
	SNNP	2	0.50
Residence	Urban	303	73.20
	Rural	111	26.80
Marital status	Single	385	93.00
	Married	20	4.80
	Divorced	9	2.20
Living	Alone	18	4.30
arrangement	With both parents	253	61.10
	With father only	47	11.40
	With mother only	59	14.30
	Other relatives	21	5.10
	With friends	5	1.20
	With husband	5	1.20
	With wife	6	1.40
Family size	≤ 3	66	15.90
	4–6	253	61.20
	≥7	95	22.90
Mothers educational	Unable to read and write	27	6.52
status	Read and write	71	17.15
	Primary school	44	10.63
	Secondary	90	21.74
	Diploma	116	28.02
	Tertiary and above	66	15.94
Fathers educational status	Unable to read and write	20	4.80
	Read and write	34	8.20
	Primary school	29	7.00
	Secondary school	76	18.40
	Diploma	80	19.30
	Tertiary and above	175	42.30
Mothers occupation	Housewife	190	45.90
	Government employed	131	31.60
	Private	34	8.20
	Merchant	57	13.80
	Daily laborer	2	0.50
Fathers occupation	Government employed	186	44.90
	Private employed	82	19.80
	Merchant	108	26.10
	Farmer	31	7.50
	Daily laborer	7	1.70
Knowing Parents' average monthly income	Yes	196	47.30
	No	218	52.70
Parents average monthly income (*n =* 196)	<1,000	3	1.50
	1,000–2,000	20	10.20
	>2,000	173	88.30
Do you get pocket money	Yes	248	59.90
	No	166	40.10
Amount of daily pocket *N =* 248	≤ 20	149	60.08
	>20	99	39.92

### Sexual Related Characteristics of Participants

A total of 229 (55.3%) study participants have had a girlfriend or boyfriend, and out of those 117 (51.1%) have had two or more boys or girlfriends. More than half of the study participants, 216 (52.2%), had sexual intercourse ever. Of those sexually active participants, about 177 (42.8%) started sex before the age of 18 years. In this finding, 161 (38.9%) of the study participants reported that they have had the first sexual intercourse with their boyfriend and or girlfriend. About 91 (35.8%) of the study participants reported to have had sex with personal desire (Table 2).

**Table 2:** Sexual practice-related characteristics of study participants in Gondar city, Northwest Ethiopia, 2019 (*n* = 414).

**Variables**		**Frequency**	**Percentage**
Having a girl/boyfriend	Yes	229	55.30
	No	185	44.70
Number of girl/boyfriend (*n =* 229)	1	112	48.90
	≥2	117	51.10
Having started sexual intercourse	Yes	216	52.20
	No	198	47.80
Age of 1st sex (*n =* 216)	<18	177	81.90
	18–24	39	18.10
Age of 1st sex with a sexual partner (*n =* 216)	Younger	30	13.90
	Older	77	35.60
	Equal age	16	7.40
	Don't know	93	43.10
With whom having sex with (*n =* 216)	Boy or girlfriend	161	74.50
	Commercial sex workers	15	6.90
	Unknown person	32	14.80
	Relatives	8	3.80
Reason to start sex (*n =* 216)	Personal desire	91	35.83
	Peer pressure	63	24.80
	Influence of alcohol	61	24.02
	Influence of chat or drug	6	2.36
	Economic problem	22	8.66
	Rape	11	4.33
Number of partners having sex with (*n =* 216)	Only one	129	60.00
	Two or more	70	32.60
	Don't remember	17	7.40
Number of intimate friends in the last 12 months	Only one	131	31.60
	Two or more	199	48.10
	Don't have	84	20.30
Sexual contact in the last 12 month	Yes	179	43.20
	No	235	56.80
Using condom during sex (*n =* 216)	Yes	127	41.00
	No	89	59.00
Frequency of using condom(*n =* 127)	Always	42	33.10
	Rarely	85	66.90
Substance usage	Yes	96	23.20
	No	318	76.80
Types of substances used (*N =* 96)	Chat	53	48.62
	Cigarettes	22	20.18
	Hashish	32	29.36
	Weeds	2	1.83
Usage of alcohol	Yes	211	51.00
	No	203	49.00
Frequency of alcohol usage (*n =* 211)	Always	22	10.43
	Some times	120	56.87
	Only on holidays	69	32.70
Enjoy night clubs	Yes	154	37.20
	No	260	62.80
Frequency of enjoying nightclubs (*n =* 154)	Daily	3	1.95
	Every weekend	44	28.57
	At least twice a week	26	16.88
	At least once a month	81	52.60
Sex with CSW (*n =* 124)	Yes	93	75.00
	No	31	25.00
Sexually transmitted infection (*n =* 216)	Yes	26	12.00
	No	190	88.00

### Psychological and Social Media Related Characteristics of Study Participants

In total, 155 (37.4%) study participants have experienced peer pressure to have had sex, while 208 (50.2%) participants have reported their best friends have had sex. The majority of the students, 370 (89.4%), have used social media, with telegram being used dominantly by 264 of them (35.9%) (Table 3).

**Table 3:** Psychological and social media-related characteristics of study participants in Gondar city, Northwest Ethiopia, 2019 (*n* = 414).

**Variables**		**Frequency**	**Percentage**
Peer pressure	Yes	155	37.40
	No	259	62.60
Best friends have had sex	Yes	148	35.80
	No	208	50.20
	Don't know	58	14.00
Number of friends who have had sex	All	76	18.40
	None	157	37.90
	Don't know	181	43.70
Academic performance	Excellent	149	36.00
	Good	214	51.70
	Poor	51	12.30
Social media used	Yes	370	89.40
	No	44	10.60
Frequency of social media used	Daily	132	35.68
	Often	97	26.22
	Occasionally	80	21.61
	Rarely	61	16.49
Type of social media used^*^	Facebook	242	32.88
	Telegram	264	35.87
	Instagram	114	15.49
	Viber	84	11.41
	IMO	27	3.67
	Whats up	5	0.68
Watching pornography	Yes	185	44.70
	No	229	55.30
Frequency of watching pornography	Daily	32	17.30
	Often	27	14.60
	Occasionally	46	24.90
	Rarely	80	43.20
With whom watching pornography	Alone	126	68.10
	Boy or girlfriend	50	27.00
	Family members	4	2.20
	Unknown person	5	2.70
What do you feel about risky sexual behavior??	It is bad	143	34.50
	It hurts us	77	18.60
	Don't know	194	46.90
Having risky sexual behavior	Yes	204	49.30
	No	210	50.70

* *More than one response noted*.

### Prevalence of Risky Sexual Practices

**Figure 1:**
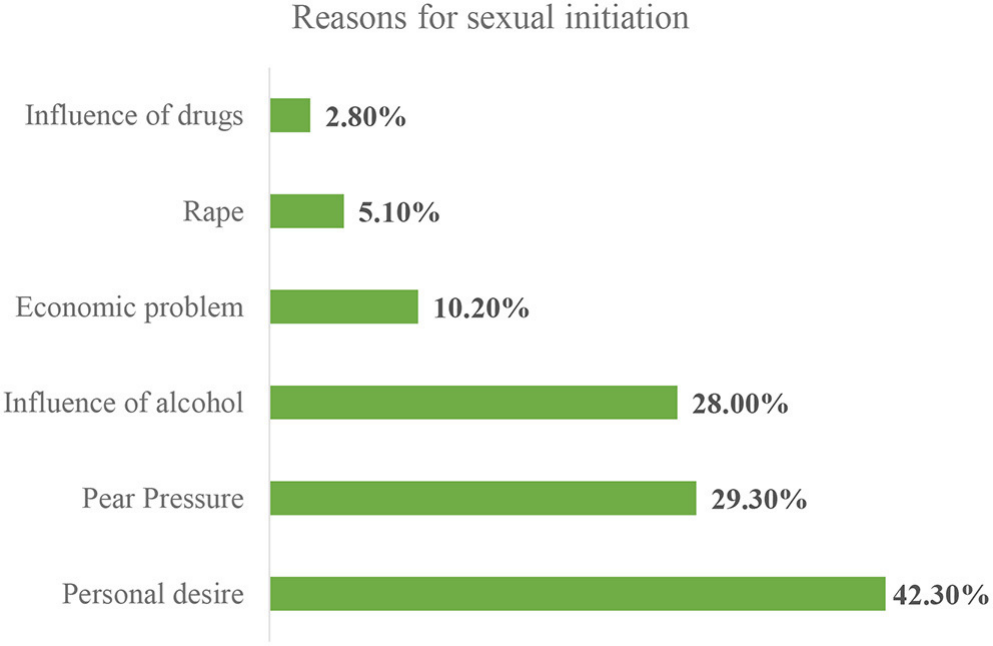
Reasons for sexual initiation among private preparatory school students in Gondar city, northwest Ethiopia, 2019.

In this study, the risky sexual practices were assessed by variables like number of multiple sexual partners, age at first sex, condom use, and sex with commercial sex workers. Thus, the prevalence of risky sexual practices among preparatory school students in Gondar city was 49.3% (95%CI: 46.4%−55.6%). Male students were highly (57.8%) engaged in risky sexual practices. This study illustrated that 52.2% of the study participants are sexually active, with 43.2% having sex 12 months before this data collection period. The predominant reason for initiation of sex was being under the influence of alcohol followed by peer pressure (Figure 1).

### Factors Associated With Risky Sexual Practices

On bivariable analysis, variables that were found to have an association with risky sexual practices were daily pocket money, peer pressure, drinking alcohol, and watching pornography. A multivariable analysis was also done to identify the independent effect of the variables by controlling the confounding effect of other variables (31). Accordingly, three variables were found to have a significant association with risky sexual behaviors at a *p-*value <0.05. These include consuming alcohol, experiencing peer pressure to have sex, and watching pornography.

The odds of having risky sexual practices among students experiencing peer pressure to have sex was nearly two times higher than those who did not experience such pressure from their peers (AOR = 1.99; 95%CI: 1.21–3.26). Moreover, this study has also revealed that exposure to pornographic movies puts the students at higher risk of practicing risky sexual practices. Students who watched pornographic movies had 2.82 times higher odds of undertaking risky sexual practices than their counterparts who did not watch pornographic movies (AOR = 2.82; 95%CI: 1.74–4.56).

This study further declared that students who have reported drinking alcohol were 4.88 times more likely to engage in risky sexual practices as compared to their counterparts who did not drink alcohol (AOR = 4.88; 95%CI: 3.06–7.79) (Table 4).

**Table 4:** Bivariable and multivariable logistic regression analysis of factors associated with risky sexual practice among preparatory school students, in Gondar city, northwest Ethiopia, 2019.

**Variables**		**Risky sexual Practice**		**COR 95%CI**	**AOR 95%CI**
		**Yes**	**No**		
Get daily pocket money	Yes	142	106	2.24 (1.5–3.36)	1.54 (0.96–2.47)
	No	62	104	1	
Drinking alcohol	Yes	152	59	7.48 (4.84–11.56)	4.88 (3.06–7.79)^**^
	No	52	151	1	
Peer pressure	Yes	107	48	3.72 (2.44–5.69)	1.99 (1.21–3.26)^*^
	No	97	162	1	
Watching pornography	Yes	130	55	2.8 (1.25–4.53)	2.82 (1.74–4.56)^**^
	No	105	124	1	

*1, Reference category,*

** *Significant at p ≤ 0.001,*

* *Significant at p < 0.05*.

## Discussion

We have conducted this study to determine the prevalence of risky sexual practices and factors associated with them among Gondar city preparatory school students. Accordingly, our study illustrated that the proportion of respondents who engaged in risky sexual practices was 49.3 % (95%CI: 46.4–55.6). The key findings of this study point out the role of social determinants of health in risky sexual practice in youths and the necessity of conducting further qualitative research to address behavioral-related social determinants of health in the context of sexuality. The findings also underpin the need for considering the results when developing and implementing strategies to improve the sexual behavior of youths as they do not usually get the right information, good overall care, and enough consultation time.

Accordingly, this finding is in line with a study conducted in Lalibela town (46.5%) (1). This similarity might be due to geographical as well as cultural closeness between the two study areas; as a result, the population's attitude toward having sex and taking safety measures would be equally affected while it is lower than a study conducted on students of Debre Markos University (58.15%) (19) and by the National Adolescent and Youth Health Strategy (2016–2020) (32). The possible difference observed might be because of the difference in educational level, living arrangement, study area, sample size differences, and unpredictability behaviors of sexual practices of youths.

On the other hand, the prevalence of risky sexual practices in this study is much higher compared to a study conducted in Gondar city administration where the overall prevalence of risky sexual practices was 12.8% (26), in Arsi Negelle was 32% (33), in Axum was 19% (3), in Wolaita Sodo was 24.7% (12), and in Humera was 13.7% (34). This variation among reports might be due to differences in the difference in grade level since our study participants are higher grade students (preparatory students) compared to the previous two studies conducted among high school students so that higher grade students had more exposure to risky sexual practices than junior high school students. Since they consider themselves to be in place to practice everything, they are much more eager to test their sexual ability than those in the lower classes.

Turning to the associated factors, respondents who watched the pornographic films were at higher risk to engage in risky sexual practice with an odds ratio of 2.82 (AOR = 2.82; 95%CI: 1.74–4.56). This may be due to the access to enhanced mobile technology, the internet, and widespread porn video media portrayals across every corner of the world which fuels the problem of risky sexual practice among youths. Regarding this, youths are highly addicted to the usage of more advanced technologies that pave the way for watching these pornographic videos that initiate them to practice without thinking about the consequences. Furthermore, youths are eager and sensitive to experiment with what they hear and look at those videos; since they are at the natural transition stage to adults and hence, they are prone to be driven by porn videos they watch to experiment with risky sex. This finding is consistent with studies in Gondar (26), Haramaya (35), Humera (34), Arba Minch (10), xum (3), Nekemte (36), and Jimma (37).

Furthermore, this study has also indicated that respondents who drink alcohol were 4.88 times (AOR = 4.88; 95%CI: 3.06–7.79) at higher risk to involve in risky sexual practices. This may be a result of the myopic effect of alcohol to make a rational decision by considering the consequence of risky sexual behavior. Individuals with alcohol influence deciding without analyzing consequences to be followed after having sex and drinking alcohol is one of the driving factors for individuals to engage in unprotected sex (inconsistent use of a condom), sex with commercial sex workers, and sex with an unknown person (multiple sexual partners) and might result in STI including HIV and unwanted pregnancy in case of women, which are all indicators of risky sexual behavior. Similar findings were also observed in studies conducted in Ethiopia (11, 38, 39), Kenya (40), and Saudi (41).

Lastly, respondents who experienced peer pressure to have sex were 1.99 times (AOR = 1.99; 95%CI: 1.21–3.26) more at risk of risky sexual practices than their counterparts. A plausible explanation is that, since youth spend most of their time with their peers, peers are the most influential socializing agent for sexuality among youths. As youths need attention and recognition from peers, they are liable to behave in a manner their intimate friend practices. So, if they have a sexually active friend that provoke them to involve in such a way, they will be in danger of committing unhealthy sexual behaviors the same way their friends do. This finding is supported by studies conducted in Gondar (26), Lalibela (6), Arsi Negelle (33), and Jimma (42).

In our study, factors such as age, sex, religious attendance, residence, living arrangement, parental educational status, having a girlfriend or boyfriend, having started sex, age of first sex, and enjoying nightclubs had not had a significant association with risky sexual behavior. Studies revealed that being in the age group 21–23 (43), being a woman (37), urban residence (1), and enjoying nightclubs (44) had its effect by increasing the likelihood of risky sexual behavior. While regular religious attendance and living with parents protect students from those risky sexual practices (34), not having such a significant association in our study in contrary with the other findings might be because of differences in the educational level of participants, sample size, the year difference the studies conducted, and because of unpredictability behaviors of sexual practices of youths.

### Limitations of the Study

Despite the self-administered data collection method, due to the sensitivity of the topic itself, social desirability bias may be introduced, which can lead to underestimating the prevalence of risky sexual practices among in-school youth students. In addition, due to the nature of the study design, it may not show causation. This study is not triangulated with a qualitative study design, which is better in in-school youths describing their life experiences and behavioral factors. Finally, since the study was conducted in a single city, it could not be generalized to the rest of the country.

### Implications of the Study

The evidence from this study calls upon policymakers, program managers, researchers, school administration, parents, and communities to play a role in preventing risky sexual behaviors and their consequences through, information, education, counseling provision, community mobilization, and integration of behavioral, social, policy, structural, or other interventions.

## Conclusion

The findings of this study strongly indicated that the prevalence of risky sexual practices among school youths in Gondar city, Ethiopia is quite common. Factors like ever using alcohol, ever watching pornographic videos, and peer pressure to have sex were important factors for increasing the magnitude of risky sexual behaviors among in-school youths. Thus, collaborative efforts are needed from parents, schools, health facilities, and the government to mitigate the exposure of in-school youths toward peer pressure, drinking alcohol, and watching pornographic films which in turn helps to bring about healthy sexual behaviors among them.

## Data Availability Statement

The original contributions presented in the study are included in the article/supplementary material, further inquiries can be directed to the corresponding author/s.

## Ethics Statement

The studies involving human participants were reviewed and approved by University of Gondar Institutional Review Board. Written informed consent to participate in this study was provided by the participants' legal guardian/next of kin.

## Author Contributions

ZAz wrote the proposal, participated in data collection, analyzed the data, drafted the article, and prepared the manuscript. LT, MW, ATades, ATadel, ZAn, and BT approved the proposal with revision, participated in data analysis, and revised subsequent drafts of the article. All the authors read and approved the final manuscript.

## Conflict of Interest

The authors declare that the research was conducted in the absence of any commercial or financial relationships that could be construed as a potential conflict of interest.

## Publisher's Note

All claims expressed in this article are solely those of the authors and do not necessarily represent those of their affiliated organizations, or those of the publisher, the editors and the reviewers. Any product that may be evaluated in this article, or claim that may be made by its manufacturer, is not guaranteed or endorsed by the publisher.

## Abbreviations

AIDS: Acquired Immune-Deficiency Syndrome
CSW: Commercial Sex Workers
HIV: Human Immunodeficiency Virus
RSB: risky sexual behaviors
STI: Sexually Transmitted Infections
STD: Sexually Transmitted Diseases
WHO: World Health Organization.
